# The impact of testing and treatment on the dynamics of Hepatitis B virus

**DOI:** 10.12688/f1000research.72865.1

**Published:** 2021-09-17

**Authors:** Olajumoke Oludoun, Olukayode Adebimpe, James Ndako, Michael Adeniyi, Oluwakemi Abiodun, Babatunde Gbadamosi

**Affiliations:** 1 Physical sciences, Landmark University, Omu Aran, Kwara State, Nigeria; 2 Physical Sciences, Landmark University, Omu Aran, Kwara, Nigeria; 3 Biological Sciences, Landmark University, Omu Aran, Kwara, Nigeria; 4 Mathematics, Lagos State Polytechnic, Lagos, Lagos, Nigeria; 5 Physical Sciences (Mathematics Programme), Landmark University, Omu Aran, Kwara, Nigeria; 6 Computer Science, Landmark University, Omu Aran, Kwara, Nigeria

**Keywords:** : Positivity and boundedness of solutions, Equilibria of solutions, Next generation matrix, Linearization, Lyapunov functions, local and global stabilities.

## Abstract

Despite the intervention of WHO on vaccination for reducing the spread of Hepatitis B Virus (HBV), there are records of the high prevalence of HBV in some regions. In this paper, a mathematical model was formulated to analyze the acquisition and transmission process of the virus with the view of identifying the possible way of reducing the menace and mitigating the risk of the virus. The models' positivity and boundedness were demonstrated using well-known theorems. Equating the differential equations to zero demonstrates the equilibria of the solutions i.e., the disease-free and endemic equilibrium. The next Generation Matrix method was used to compute the basic reproduction number for the models. Local and global stabilities of the models were shown via linearization and Lyapunov function methods respectively. The importance of testing and treatment on the dynamics of HBV were fully discussed in this paper. It was discovered that testing at the acute stage of the virus and chronic unaware state helps in better management of the virus.

## 1. Introduction

Hepatitis is an inflammation/scarring of the liver that contributes to various health complications, including death. It occurs due to an immune system attack by the virus in the liver and damages this vital organ of the body in the process ( Ciupe *et al.,* 2014). The hepatitis B virus (HBV) can survive outside the body for at least seven days. If the virus enters the body of someone who is not protected by vaccination during this time, it can still cause infection. The average incubation time for HBV is 75 days, but it can range from 30 to 180 days. Within 30 to 60 days of infection, the virus may be detected, persist, and grow into chronic hepatitis B ( CDC, 2019). Hepatitis B is most common in the Western Pacific region with prevalence rate 6.2% and Africa with prevalence rate of 6.1%, with the Americas region (0.7%) having the lowest prevalence ( WHO, 2019).

In highly endemic areas, the most common form of transmission of hepatitis B is from mother to child at birth (vertical transmission) or through horizontal route (contact with infected blood), particularly from infected children to uninfected children during the first five years of life. Chronic infection develops in infants infected by their mothers or before the age of five. It is often transmitted through transdermal or mucosal contact of infected persons to infected blood and different body fluids, such as spittle, catamenial, vaginal and spermatic fluids and, to a lesser degree, perspiration, breast milk, tears, and urine. In particular, hepatitis B can be transmitted through sexual contact in unvaccinated men who have sex with men (MSM) and heterosexual people who have multiple sexual partners or have contact with sex workers. However, adult infection contributes to chronic hepatitis in less than 5% of cases. This transmission may similarly ensue when needles and syringes are reused, whether in healthcare settings or among drug users. Furthermore, an infection can occur during medical, surgical, and dental procedures, such as tattooing or using razors and other similar objects contaminated with infected blood ( Mpeshe and Nyerere, 2019).

## 2. Mathematical formulation

Some chronic carriers are unaware of their status and as such transmit the virus unknowingly and also at higher risk of cirrhosis and makes treatment less effective ( Niederau, 2014, Mcpherson *et al.,* 2013, Cohen *et al.,* 2011, Piorkowsky, 2009, Lin *et al.,* 2009, Meffre *et al.,* 2004).

In view of this, this model is developed to factor the aforementioned set of people. In the model, the population is divided into the following different groups: the susceptible, the acute, the chronic unaware carriers, the chronic aware carriers, the treated chronic aware and the recovered individuals.

The total population at time t, denoted by $N\left(t\right)$ is divided into the six subgroups corresponding to different epidemiological status: susceptible individuals $\;S\left(t\right)$ , acute $A\left(t\right)$ , unaware chronically infected $C_{u}\left(t\right)$ , aware chronically infected $C_{a}\left(t\right)$ , treated $T_{c}\left(t\right)$ , and removed/recovered class $R\left(t\right)$ . The model equation is subject to the initial conditions,


$$S\left(t\right)\geq 0,A\left(t\right)\geq 0,C_{u}\left(t\right)\geq 0,C_{a}\left(t\right)\geq 0,T_{c}\left(t\right)\geq 0,R\left(t\right)\geq 0$$ (1)

**Figure 1.**
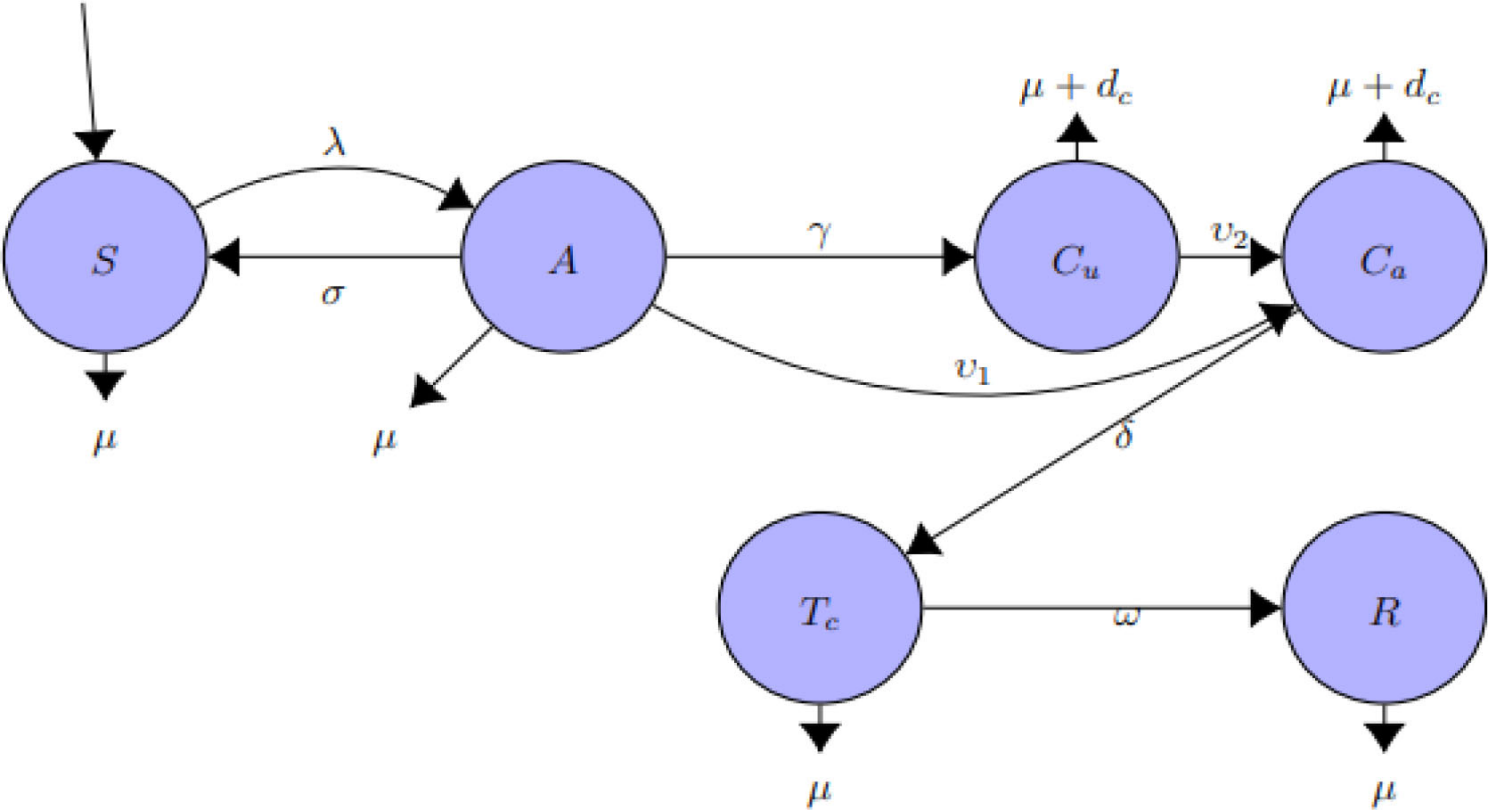
Compartmental flow diagram of HBV model.

Figure 1 represents schematically the epidemiology of HBV infected model. The different disease stages are reproduced by the different circle and the arrows indicate the way individual progress from one stage to the other. It is assumed that at time, t, susceptible individuals, S, enter the population at a constant rate, Π. For all classes, individuals die at a constant natural mortality rate, μ. HBV chronically infected individuals $(C_{u}\left(t\right)$ , $C_{a}\left(t\right))\;$ have an additional death rate due to HBV, ${\mathrm{d}}_{c}$ (Zhang and Zhang (2018)). It is assumed that HBV infected individuals on treatment, $T_{c}\left(t\right)$ do not transmit HBV infection. Susceptible individuals, $S\left(t\right),$ may acquire HBV infection when in contact with individuals in A $,C_{u},$ and $\;C_{a},$ populace at a rate, λ (force of infection associated with HBV), where

$$\lambda =\frac{\beta \left(A+\alpha_{1}C_{u}+\alpha_{2}C_{a}\right)}{N}$$ (2)

Parameter β represents the probability that a contact will result in an HBV infection while $\alpha_{1},\alpha_{2}>1$ respectively account for modification parameter of chronic HBV-infected individuals.

A proportion of the acute HBV-infected individuals, σ, spontaneously clear the virus, then return to being susceptible. The HBV acutely infected individuals develop to chronic without been aware if no testing at a rate, γ. The acutely infected and chronic unaware individual progress to chronic aware stage with a testing $\nu_{1},\nu_{2}$ respectively and moved to treatment stage after testing at the rate δ . ω is the recovery rate of treated infected individual with full immunity.

These assumptions lead to the system of equations in (3)

$$\frac{dS}{dt}=\Pi -\lambda S+\sigma A-\mu S\frac{dA}{dt}=\lambda S-\left(\sigma +\gamma +\nu_{1}\right)A\frac{dC_{u}}{dt}=\gamma A-\left(\nu_{2}+\mu +d_{c}\right)C_{u}\frac{dC_{a}}{dt}=\nu_{2}C_{u}+\nu_{1}A-\left(\delta +\mu +d_{c}\right)C_{a}\frac{dT_{c}}{dt}=\delta C_{a}-\left(\omega +\mu \right)T_{c}\frac{dR}{dt}=\omega T_{c}-\mu R$$ (3)

where $\lambda =\frac{\beta \left(A+\alpha_{1}C_{u}+\alpha_{2}C_{a}\right)}{N}$

### 2.1 Positivity and boundedness of solutions

For the system of equations (3) to be epidemiologically meaningful, it is important to prove that all solution with non-negative initial conditions will remain non-negative.

**Lemma 1:** The initial values of the parameters are

$$\left\{S\left(0\right)\geq 0,A\left(0\right)\geq 0,C_{u}\left(0\right)\geq 0,C_{a}\left(0\right)\geq 0,T_{c}\left(0\right)\geq 0,R\left(0\right)\geq ,0\text{and}N\left(0\right)\geq 0\right\}\in \Phi$$

Then the solution of the model $\left\{S\left(t\right),A\left(t\right),C_{u}\left(t\right),C_{a}\left(t\right),T_{c}\left(t\right),R\left(t\right),N\left(t\right)\right\}$ is positive for all t≥0.

**Proof**

Considering the first equation in (3),

$$\frac{\mathrm{dS}}{\mathrm{dt}}=\Pi -\mathrm{\lambda S}+\mathrm{\sigma A}-\mathrm{\mu S}$$

$$\frac{\mathrm{dS}}{\mathrm{dt}}\geq -\left(\lambda +\mu \right)S\int \frac{1}{S}\mathrm{dS}\geq \int -\left(\lambda +\mu \right)\mathrm{dt}$$

$$S\geq S_{0}e^{-\left(\lambda +\mu \right)t}\geq 0$$

Hence, S≥0

with respect to the second equation in (3);

$$\frac{\mathrm{dA}}{\mathrm{dt}}=\mathrm{\lambda S}-\left(\sigma +\gamma +\nu_{1}\right)A$$

$$\frac{\mathrm{dA}}{\mathrm{dt}}\geq -\left(\sigma +\gamma +\nu_{1}\right)A$$

$$\int \frac{1}{A}\mathrm{dA}\geq \int -\left(\sigma +\gamma +\nu_{1}\right)\mathrm{dt}$$

$$A\geq A_{0}e^{-\left(\sigma +\gamma +\nu_{1}\right)t}\geq 0$$

Hence, A≥0 . Same goes for the other compartments

Clearly, the above state variables are positive on bounding plane ${\mathbb{R}}_{+}^{6}$ .

For the boundedness the following calculation follows:

$$N\left(t\right)=S\left(t\right)+A\left(t\right)+C_{u}\left(t\right)+C_{a}\left(t\right)+T_{c}\left(t\right)+R\left(t\right)$$

$$N^{\prime }=S\prime +A\prime +C_{u}\prime +C_{a}\prime +T_{c}\prime +R\prime$$

$$N^{\prime }=\Pi -\mathrm{\lambda S}+\mathrm{\sigma A}-\mathrm{\mu S}+\mathrm{\lambda S}-\left(\sigma +\gamma +\nu_{1}\right)A+\mathrm{\gamma A}-\left(\nu_{2}+\mu +d_{c}\right)C_{u}+\nu_{2}C_{u}+\nu_{1}A-\left(\delta +\mu +d_{c}\right)C_{a}+\delta C_{a}-\left(\omega +\mu \right)T_{c}+\omega T_{c}-\mathrm{\mu R}$$ (4)

Simplifying:

$$N^{\prime }+\mathrm{\mu N}=\Pi -d_{c}C_{u}$$ (5)

$$N^{\prime }+\mathrm{\mu N}\leq \Pi$$ (6)

Integrating gives:

$$N^{\prime }\leq \frac{\Pi }{\mu }+ke^{-\mathrm{\mu t}}$$

$$\max_{\lim_{n\to \infty }}N\leq \lim_{n\to \infty }\left(\frac{\Pi }{\mu }+ke^{-\mathrm{\mu t}}\right)\leq \frac{\Pi }{\mu }$$

It follows that the solutions of the model system (3) are positive and bounded in the region

$$T=\left\{\left(S+A+C_{u}+C_{a}+T_{c}+R\right)\right\}\in {\mathbb{R}}_{+}^{6}:S+A+C_{u}+C_{a}+T_{c}+R\leq \frac{\Pi }{\mu }$$

It follows from Lemma 1 that it is sufficient to consider the dynamics of system (3) and the model can be considered to be epidemiologically well-posed.

### 2.2 Equilibrium points

The disease-free equilibrium of the equation (3) exists and is given by:

$$\left(E_{0}\right)=\left[\frac{\Pi }{\mu },0,0,0,0,0\right]$$ (7)

The endemic steady states are calculated here which is done by setting system of equation in (3.3.3) to zero and setting $S=S^{*},A=A^{*},C_{u}={C_{u}}^{*},C_{a}={C_{a}}^{*},T_{c}={T_{c}}^{*},R=R^{*}$ so that

$$S^{*}=\left(\frac{\begin{array}{l} \Pi (\mu^{3}+\left(\delta +\gamma +2d_{c}+\upsilon_{1}+\upsilon_{2}\right)\mu^{2}+({d_{c}}^{2}+\left(\upsilon_{1}+\upsilon_{2}+\delta +\gamma \right)d_{c}+\left(\upsilon_{2}+\delta \right)\gamma +\left(\upsilon_{2}+\delta \right)\upsilon_{1}+\delta \upsilon_{2})\mu )\\ +\delta \left(\upsilon_{1}d_{c}+\upsilon_{2}\left(\upsilon_{1}+\gamma \right)\right)\left(\upsilon_{1}+\sigma +\gamma \right) \end{array}}{L}\right)$$ (8)

$$A^{*}=-\left(\frac{\begin{array}{c} \left(\upsilon_{1}-\beta +\gamma +\sigma \right){d_{c}}^{2}+\left(\left(2\upsilon_{1}-2\beta +2\gamma +2\sigma \right)\mu +\left(\upsilon_{2}+\delta -\beta \alpha_{1}\right)\gamma +\left(\upsilon_{2}+\delta -\beta \alpha_{2}\right)\upsilon_{1}-\left(\beta -\sigma \right)\left(\upsilon_{2}+\delta \right)\right)d_{c}\\ +\left(\upsilon_{1}-\beta +\gamma +\sigma \right)\mu^{2}+\left(\left(\upsilon_{2}+\delta -\beta \alpha_{1}\right)\gamma +\left(\upsilon_{2}+\delta -\beta \alpha_{2}\right)\upsilon_{1}-\left(\beta -\sigma \right)\left(\upsilon_{2}+\delta \right)\right)\mu +\left(\left(-{\delta \alpha }_{1}-\alpha_{2}\upsilon_{2}\right)\beta \right)\Pi \end{array}}{L}\right)$$ (9)

$${C_{u}}^{*}=A^{*}\gamma \;$$ (10)

$${C_{a}}^{*}=\frac{{C_{u}}^{*}((\upsilon_{1}+\upsilon_{1}\mu )+\upsilon_{2}\left(\upsilon_{1}+\gamma \right))}{\gamma }$$ (11)

$${T_{c}}^{*}=-\left({C_{a}}^{*}\left(\frac{\mu^{2}}{{d_{c}}^{2}}+\frac{\mu }{d_{c}}+\frac{\upsilon_{2}}{\mu^{2}}+\frac{1}{\upsilon_{1}+\upsilon_{2}}\right)\delta \right)$$ (12)

$$R^{*}=\omega {T_{c}}^{*}$$ (13)

where

$$L=\left(\left(\upsilon_{2}+\mu +d_{c}\right)\left(\upsilon_{1}+\gamma +\sigma \right)\mu^{3}+\left(\left(2\upsilon_{1}+2\gamma +2\sigma \right)d_{c}\right)+\left(\upsilon_{1}+\gamma +\sigma \right)\upsilon_{2}+\left(\beta +\delta \right)\gamma +\left(\beta +\delta \right)\upsilon_{1}+\delta \sigma \right)\mu^{2}+\left(\left(\upsilon_{1}+\gamma +\sigma \right){d_{c}}^{2}+\left(\left(\upsilon_{1}+\gamma +\sigma \right)\upsilon_{2}-\gamma^{2}+\left(2\beta +\delta -\sigma -2\upsilon_{1}\right)\gamma -\upsilon_{1}^{2}+\left(2\beta +\delta -\sigma \right)\upsilon_{1}+\delta \sigma \right)d_{c}+\left(\left(\beta +\delta \right)\gamma +\left(\beta +\delta \right)\upsilon_{1}+\delta \sigma \right)\upsilon_{2}+\beta \left(\upsilon_{1}+\gamma \right)\left(\gamma \alpha_{1}+\alpha_{2}\upsilon_{1}+\delta \right)\right)\mu -\left(\upsilon_{1}+\gamma \right)\left(\upsilon_{1}-\beta +\gamma +\sigma \right){d_{c}}^{2}+\left(-\left(\upsilon_{1}+\gamma \right)\left(\upsilon_{1}-\beta +\gamma +\sigma \right)\upsilon_{2}+\left(\beta \alpha_{1}-\delta \right)\gamma^{2}+\left(\left(-\delta +\left(\alpha_{1}+\alpha_{2}\right)\beta \right)\upsilon_{1}+\delta \left(\beta -\sigma \right)\right)\gamma +\left(\delta +\alpha_{2}\upsilon_{1}\right)\beta \upsilon_{1}\right)d_{c}+\beta \left(\gamma \alpha_{1}+\alpha_{2}\upsilon_{1}+\delta \right)\upsilon_{2}+\delta \gamma \alpha_{1})\left(\upsilon_{1}+\gamma \right)\left(\delta +\mu +d_{c}\right)\left(\mu +\omega \right)$$

### 2.3 Basic reproduction number

The basic reproduction number ( $R_{o}$ ) which is the number of secondary infections caused by an infectious individual is determined by the next generation matrix which is given by $\rho \left(FV^{-1}\right)$

where:

$$F=\left[\begin{array}{ccc} \beta & \beta \alpha_{1} & \beta \alpha_{2}\\ 0 & 0 & 0\\ 0 & 0 & 0 \end{array}\right]$$

$$V=\left[\begin{array}{ccc} \sigma +\gamma +\upsilon_{1} & 0 & 0\\ -\gamma & d_{c}+\mu +\upsilon_{2} & 0\\ -\upsilon_{1} & -\upsilon_{2} & d_{c}+\mu +\delta \end{array}\right]$$

$$V^{-1}=\left[\begin{array}{ccc} \frac{1}{\sigma +\gamma +\upsilon_{1}} & 0 & 0\\ \frac{\gamma }{\left(\sigma +\gamma +\upsilon_{1}\right)\left(d_{c}+\mu +\upsilon_{2}\right)} & \frac{1}{d_{c}+\mu +\upsilon_{2}} & 0\\ \frac{\gamma \upsilon_{2}+\upsilon_{1}\mu +\upsilon_{1}d_{c}+\upsilon_{1}\upsilon_{2}}{\left(\sigma +\gamma +\upsilon_{1}\right)\left(d_{c}+\mu +\upsilon_{2}\right)\left(d_{c}+\mu +\delta \right)} & \frac{\upsilon_{2}}{\left(d_{c}+\mu +\upsilon_{2}\right)\left(d_{c}+\mu +\delta \right)} & \frac{1}{\left(d_{c}+\mu +\delta \right)} \end{array}\right]$$

$$R_{o}=\frac{\beta }{\sigma +\gamma +\upsilon_{1}}+\frac{\beta \alpha_{1}\gamma }{\left(\sigma +\gamma +\upsilon_{1}\right)\left(d_{c}+\mu +\upsilon_{2}\right)}+\frac{\beta \alpha_{2}\left(\gamma \upsilon_{2}+\upsilon_{1}\mu +\upsilon_{1}d_{c}+\upsilon_{1}\upsilon_{2}\right)}{\left(\sigma +\gamma +\upsilon_{1}\right)\left(d_{c}+\mu +\upsilon_{2}\right)\left(d_{c}+\mu +\delta \right)\;}$$ (14)

### 2.4 Local stability analysis of the disease-free equilibrium $E_{0}$

**Theorem 1**: $E_{0}$ is locally asymptotically stable if $R_{0}$ < 1 and unstable if $R_{0}$ > 1.

**Proof:** The resulting matrix from the linearized model is $\frac{\mathrm{dX}}{\mathrm{dt}}=\mathrm{AX}$

$$X={\left(x_{1},x_{2},x_{3},x_{4},x_{5,}x_{6}\right)}^{T},\left(x_{1},x_{2},x_{3},x_{4},x_{5,}x_{6}\right)\in R_{+}^{6},\text{and}$$

The resulting Jacobian matrix at $E_{0}$ is

$$J\left(E_{0}\right)=\left[\begin{array}{llllll} -\mu -\lambda & -\beta +\sigma & -\beta \alpha_{1} & -\beta \alpha_{2} & 0 & 0\\ 0 & \beta -\sigma -\gamma -\nu_{1}-\lambda & \beta \alpha_{1} & \beta \alpha_{2} & 0 & 0\\ 0 & \gamma & -d_{c}-\mu -\nu_{2}-\lambda & 0 & 0 & 0\\ 0 & \nu_{1} & \nu_{2} & -d_{c}-\mu -\delta -\lambda & 0 & 0\\ 0 & 0 & 0 & \delta & -\omega -\mu -\lambda & 0\\ 0 & 0 & 0 & 0 & \omega & -\mu -\lambda \end{array}\right]$$ (15)

From (15), $\lambda_{1}=-\mu ,\lambda_{2}=-\omega -\mu ,\;\lambda_{3}=-\mu$

and the resulting quadratic equation is:

$$\left(\beta -\sigma -\gamma -\nu_{1}-\lambda \right)\left(-d_{c}-\mu -\nu_{2}-\lambda \right)\left(-d_{c}-\mu -\delta -\lambda \right)-\beta \alpha_{1}\left(-d_{c}-\mu -\delta -\lambda \right)\gamma +\beta \alpha_{2}\left(\left(-d_{c}-\mu -\nu_{2}-\lambda \right)\nu_{1}-\gamma \nu_{2}\right)$$ (16)

$$f\left(\lambda \right)=\lambda^{3}+\left(2\mu +\nu_{1}+\nu_{2}-\beta +\delta +\gamma +\sigma +2d_{c}\right)\lambda^{2}+\left(\beta \alpha_{2}\nu_{1}-\beta \delta -2\beta \mu -2\beta d_{c}-\beta \nu_{2}+\delta \gamma +\delta \mu +\delta \sigma +\delta d_{c}+\delta \nu_{1}+\delta \nu_{2}+2\gamma \mu +2\gamma d_{c}+\gamma \nu_{2}+\mu^{2}+2\mu \sigma +2\mu d_{c}+2\mu \nu_{1}+\mu \nu_{2}+2\sigma d_{c}+\sigma \nu_{2}+{d_{c}}^{2}+2d_{c}\nu_{1}+d_{c}\nu_{2}+\nu_{1}\nu_{2}-\gamma \beta \alpha_{1}\right)\lambda +\gamma \mu^{2}+\gamma {d_{c}}^{2}+\mu^{2}\sigma +\mu^{2}\nu_{1}+\sigma {d_{c}}^{2}+{d_{c}}^{2}\nu_{1}+\mu \sigma \nu_{2}+2\mu d_{c}\nu_{1}+\mu \nu_{1}\nu_{2}+\sigma d_{c}\nu_{2}+d_{c}\nu_{1}\nu_{2}-\beta \delta \mu -\beta \delta d_{c}-\beta \delta \nu_{2}-2\beta \mu d_{c}-\beta \mu \nu_{2}-\beta d_{c}\nu_{2}-\beta \mu^{2}-\beta {d_{c}}^{2}+\delta \gamma \mu +\delta \gamma d_{c}+\delta \gamma \nu_{2}+\delta \mu \sigma +\delta \mu \nu_{1}+\delta \sigma d_{c}+\delta \sigma \nu_{2}+\delta d_{c}\nu_{1}+\delta \nu_{1}\nu_{2}+2\gamma \mu d_{c}+\gamma \mu \nu_{2}+\gamma d_{c}\nu_{2}+2\mu \sigma d_{c}-\beta \delta \gamma \alpha_{1}-\beta \gamma \mu \alpha_{1}-\beta \gamma \alpha_{1}d_{c}+\nu_{2}\gamma \beta \alpha_{2}+\nu_{1}\beta \alpha_{2}d_{c}+\nu_{1}\beta \alpha_{2}\mu +\nu_{1}\beta \alpha_{2}\nu_{2}$$ (17)

Now, $\lambda_{1}$ , $\lambda_{2},\lambda_{3}$ < 0 since the values are assumed positive. If $R_{0}$ < 1, $E_{0}$ is stable and unstable when $R_{0}$ < 1.

### 2.5 Global stability of the disease-free equilibrium

The global behavior of the equilibrium system (3) is analyzed here in this section.

**Theorem 2:** For system (3), the disease-free equilibrium $E_{0}$ is asymptotically stable globally if $R_{0}<1$ .

**Proof:** Considering the Lyapunov function defined as:

$$G\left(A,C_{u},C_{a}\right)=\left(\frac{1}{B_{0}}\right)A+\left(\frac{\beta \alpha_{1}}{B_{0}B_{1}}+\frac{\beta \alpha_{2}\upsilon_{2}}{B_{0}B_{1}B_{2}}\right)C_{u}+\left(\frac{\beta \alpha_{2}}{B_{0}B_{2}}\right)C_{a}$$ (18)

$$G\prime \left(A,C_{u},C_{a}\right)=\left(\frac{1}{B_{0}}\right)A\prime +\left(\frac{\beta \alpha_{1}}{B_{0}B_{1}}+\frac{\beta \alpha_{2}\upsilon_{2}}{B_{0}B_{1}B_{2}}\right)C_{u}\prime +\left(\frac{\beta \alpha_{2}}{B_{0}B_{2}}\right)C_{a}\prime$$ (19)

$$G\prime \left(A,C_{u},C_{a}\right)=\left(\frac{1}{B_{0}}\right)\left(\left(\frac{\beta \left(A+\alpha_{1}C_{u}+\alpha_{2}C_{a}\right)}{N}\right)S-\left(\sigma +\gamma +\nu_{1}\right)A\right)+\left(\frac{\beta \alpha_{1}}{B_{0}B_{1}}+\frac{\beta \alpha_{2}\upsilon_{2}}{B_{0}B_{1}B_{2}}\right)\left(\mathrm{\gamma A}-\left(\nu_{2}+\mu +d_{c}\right)C_{u}\right)+\left(\frac{\beta \alpha_{2}}{B_{0}B_{2}}\right)\left(\nu_{2}C_{u}+\nu_{1}A-\left(\delta +\mu +d_{c}\right)C_{a}\right)$$ (20)

At DFE, S=N so that (20) becomes:

$$G\prime \left(A,C_{u},C_{a}\right)=\left(\frac{1}{B_{0}}\right)\left(\beta \left(A+\alpha_{1}C_{u}+\alpha_{2}C_{a}\right)-\left(\sigma +\gamma +\nu_{1}\right)A\right)+\left(\frac{\beta \alpha_{1}}{B_{0}B_{1}}+\frac{\beta \alpha_{2}\upsilon_{2}}{B_{0}B_{1}B_{2}}\right)\left(\mathrm{\gamma A}-\left(\nu_{2}+\mu +d_{c}\right)C_{u}\right)+\left(\frac{\beta \alpha_{2}}{B_{0}B_{2}}\right)\left(\nu_{2}C_{u}+\nu_{1}A-\left(\delta +\mu +d_{c}\right)C_{a}\right)\;$$ (21)

Expanding and simplifying (21) gives:

$$G\prime =\left[\frac{\beta }{B_{0}}+\frac{\beta \alpha_{1}\gamma }{B_{0}B_{1}}+\frac{\beta \alpha_{2}\upsilon_{2}\gamma }{B_{0}B_{1}B_{2}}+\frac{\beta \alpha_{2}\nu_{1}}{B_{0}B_{2}}-1\right]A+\left[\frac{\beta \alpha_{1}}{B_{0}}-\frac{\beta \alpha_{1}B_{1}}{B_{0}B_{1}}-\frac{\beta \alpha_{2}\upsilon_{2}B_{1}}{B_{0}B_{1}B_{2}}+\frac{\beta \alpha_{2}\nu_{2}}{B_{0}B_{2}}\right]C_{u}+\left[\frac{\beta \alpha_{2}}{B_{0}}-\frac{\beta \alpha_{2}B_{2}}{B_{0}B_{2}}\right]C_{a}$$ (22)

$$G\prime =\left[R_{0}-1\right]\;A\leq 0$$ (23)

From Equation (23), it can be deduced that the DFE is globally stable since $R_{0}$ < 1.

### 2.6 Local stability of endemic equilibrium

**Theorem 3:** If $R_{0}>1$ , then the endemic equilibrium is locally asymptotically stable.

**Proof:**

The endemic equilibria of system (3), denoted by $\left(S^{*},A^{*},{C_{u}}^{*},{C_{a}}^{*},{T_{c}}^{*},R^{*}\right),$ can be rewritten as:

$$\text{Let}S=x+S^{*},A=y+A^{*},C_{u}=z+{C_{u}}^{*},C_{a}=h+{C_{a}}^{*},T_{c}=p+{T_{c}}^{*},R=j+R^{*}$$

$$J=\left[\begin{array}{llllll} B_{0}-\mu -\lambda & -B_{1}+\sigma & -B_{2} & -B_{3} & B_{4} & B_{5}\\ -B_{6} & B_{7}-\sigma -\gamma -\nu_{1}-\lambda & B_{8} & B_{9} & -B_{11} & -B_{12}\\ 0 & \gamma & -d_{c}-\mu -\nu_{2}-\lambda & 0 & 0 & 0\\ 0 & \nu_{1} & \nu_{2} & -d_{c}-\mu -\delta -\lambda & 0 & 0\\ 0 & 0 & 0 & \delta & -\omega -\mu -\lambda & 0\\ 0 & 0 & 0 & 0 & \omega & -\mu -\lambda \end{array}\right]$$ (25)

From (25), $\lambda_{1}=-\mu ,\lambda_{2}=-\left(\omega +\mu \right),\lambda_{3}=-\left(d_{c}+\mu +\nu_{2}\right)$ , then;

$$J=\left[\begin{array}{lll} B_{0}-\mu -\lambda & -B_{1}+\sigma & -B_{2}\\ -B_{6} & B_{7}-\sigma -\gamma -\nu_{1}-\lambda & B_{8}\\ 0 & \gamma & -d_{c}-\mu -\nu_{2}-\lambda \end{array}\right]$$ (26)

from (26);

$$\lambda^{3}+\left(\gamma +2\mu +\sigma -B_{0}-B_{4}+d_{c}+\nu_{1}+\nu_{2}\right)\lambda^{2}+\left(2\gamma \mu -B_{0}\gamma +\gamma d_{c}+\gamma \nu_{2}+\mu^{2}+2\mu \sigma -{\mathrm{\mu B}}_{0}-2\mu B_{4}+\mu d_{c}+2\mu \nu_{1}+{\mu \nu }_{2}-\sigma B_{0}+\sigma B_{3}+\sigma d_{c}+\sigma \nu_{2}+B_{0}B_{4}-B_{0}d_{c}-B_{0}\nu_{1}-B_{0}\nu_{2}-B_{3}B_{1}-B_{4}d_{c}-B_{4}\nu_{2}+d_{c}\nu_{1}+\nu_{1}\nu_{2}\right)\lambda +B_{5}\gamma +\gamma \mu \nu_{2}+{\mu \sigma \nu }_{2}+\mu \nu_{1}\nu_{2}+B_{3}B_{2}\gamma +\gamma \mu^{2}+\mu^{2}\sigma +\mu^{2}\nu_{1}+\mu d_{c}\nu_{1}+\gamma \mu d_{c}+\mu \sigma d_{c}-\mu^{2}B_{4}-\gamma \mu B_{0}-\gamma B_{0}d_{c}-\gamma B_{0}\nu_{2}-\mu {\mathrm{\sigma B}}_{0}+\mu {\mathrm{\sigma B}}_{3}+\mu B_{0}B_{4}-\mu B_{0}\nu_{1}-\mu B_{3}B_{1}-\mu B_{4}d_{c}-\mu B_{4}\nu_{2}-\sigma B_{0}d_{c}-\sigma B_{0}\nu_{2}+\sigma B_{3}d_{c}+\sigma B_{3}\nu_{2}+B_{0}B_{4}d_{c}+B_{0}B_{4}\nu_{2}-B_{0}d_{c}\nu_{1}-B_{0}\nu_{1}\nu_{2}-B_{3}B_{1}d_{c}-B_{3}B_{1}\nu_{2}$$

The result of the determinant of the Jacobian matrix is of the form:

$$a_{0}\lambda^{3}+a_{1}\lambda^{2}+a_{2}\lambda +a_{3}$$ (27)

where

$$a_{0}=1$$

$$a_{1}=\gamma +2\mu +\sigma -B_{0}-B_{4}+d_{c}+\nu_{1}+\nu_{2}$$

$$a_{2}=2\gamma \mu -B_{0}\gamma +\gamma d_{c}+\gamma \nu_{2}+\mu^{2}+2\mu \sigma -{\mathrm{\mu B}}_{0}-2\mu B_{4}+\mu d_{c}+2\mu \nu_{1}+{\mu \nu }_{2}-\sigma B_{0}+\sigma B_{3}+\sigma d_{c}+\sigma \nu_{2}+B_{0}B_{4}-B_{0}d_{c}-B_{0}\nu_{1}-B_{0}\nu_{2}-B_{3}B_{1}-B_{4}d_{c}-B_{4}\nu_{2}+d_{c}\nu_{1}+\nu_{1}\nu_{2}$$

$$a_{3}=B_{5}\gamma +\gamma \mu \nu_{2}+{\mu \sigma \nu }_{2}+\mu \nu_{1}\nu_{2}+B_{3}B_{2}\gamma +\gamma \mu^{2}+\mu^{2}\sigma +\mu^{2}\nu_{1}+\mu d_{c}\nu_{1}+\gamma \mu d_{c}+\mu \sigma d_{c}-\mu^{2}B_{4}-\gamma \mu B_{0}-\gamma B_{0}d_{c}-\gamma B_{0}\nu_{2}-\mu {\mathrm{\sigma B}}_{0}+\mu {\mathrm{\sigma B}}_{3}+\mu B_{0}B_{4}-\mu B_{0}\nu_{1}-\mu B_{3}B_{1}-\mu B_{4}d_{c}-\mu B_{4}\nu_{2}-\sigma B_{0}d_{c}-\sigma B_{0}\nu_{2}+\sigma B_{3}d_{c}+\sigma B_{3}\nu_{2}+B_{0}B_{4}d_{c}+B_{0}B_{4}\nu_{2}-B_{0}d_{c}\nu_{1}-B_{0}\nu_{1}\nu_{2}-B_{3}B_{1}d_{c}-B_{3}B_{1}\nu_{2}$$

By the Routh–Hurwitz criterion governing the polynomials of order 3, we have the following:

1.
$a_{2}.a_{3}$ are positive
2.
$a_{1}a_{2}>a_{3}$

From equation (27), 1 and 2 are satisfied.

Therefore, endemic equilibrium is locally asymptotically stable.

### 2.7 Global stability of the endemic equilibrium

**Theorem 4:** The equations of the model have a positive distinctive endemic equilibrium whenever $R_{0}$ > 1, which is said to be globally asymptotically stable.

**Proof:** Considering the Lyapunov function defined as:

$$L\left(S^{*},A^{*},{C_{u}}^{*},{C_{a}}^{*},{T_{c}}^{*},R^{*}\right)=\left(S-S^{*}\ln\left(\frac{S}{S^{*}}\right)\right)+\left(A-A^{*}\ln\left(\frac{A}{A^{*}}\right)\right)+\left(C_{u}-{C_{u}}^{*}\ln\left(\frac{C_{u}}{{C_{u}}^{*}}\right)\right)+\left(C_{a}-{C_{a}}^{*}\ln\left(\frac{C_{a}}{{C_{a}}^{*}}\right)\right)+\left(T_{c}-{T_{c}}^{*}\ln\left(\frac{T_{c}}{{T_{c}}^{*}}\right)\right)+\left(R-R^{*}\ln\left(\frac{R}{R^{*}}\right)\right)$$ (28)

where *L* takes it derivative along the system directly as:

$$\frac{\mathrm{dL}}{\mathrm{dt}}=\left(1-\frac{S^{*}}{S}\right)\frac{\mathrm{dS}}{\mathrm{dt}}+\left(1-\frac{A^{*}}{A}\right)\frac{\mathrm{dA}}{\mathrm{dt}}+\left(1-\frac{{C_{u}}^{*}}{C_{u}}\right)\frac{dC_{u}}{\mathrm{dt}}+\left(1-\frac{{C_{a}}^{*}}{C_{a}}\right)\frac{dC_{a}}{\mathrm{dt}}+\left(1-\frac{{T_{c}}^{*}}{T_{c}}\right)\frac{dT_{c}}{\mathrm{dt}}+\left(1-\frac{R^{*}}{R}\right)\frac{\mathrm{dR}}{\mathrm{dt}}$$ (29)

$$\frac{\mathrm{dL}}{\mathrm{dt}}=\left(1-\frac{S^{*}}{S}\right)\left[\Pi -\left(\frac{\beta \left(A+\alpha_{1}C_{u}+\alpha_{2}C_{a}\right)}{N}\right)S+\mathrm{\sigma A}-\mathrm{\mu S}\right]+\left(1-\frac{A^{*}}{A}\right)\left[\left(\frac{\beta \left(A+\alpha_{1}C_{u}+\alpha_{2}C_{a}\right)}{N}\right)S-\left(\sigma +\gamma +\nu_{1}\right)A\right]+\left(1-\frac{{C_{u}}^{*}}{C_{u}}\right)\left[\mathrm{\gamma A}-\left(\nu_{2}+\mu +d_{c}\right)C_{u}\right]+\left(1-\frac{{C_{a}}^{*}}{C_{a}}\right)\left[\nu_{2}C_{u}+\nu_{1}A-\left(\delta +\mu +d_{c}\right)C_{a}\right]+\left(1-\frac{{T_{c}}^{*}}{T_{c}}\right)\left[\delta C_{a}-\left(\omega +\mu \right)T_{c}\right]+\left(1-\frac{R^{*}}{R}\right)\left[\omega T_{c}-\mathrm{\mu R}\right]$$ (30)

At equilibrium,

$$\Pi =\left(\frac{\beta \left(A*+\alpha_{1}C_{u}*+\alpha_{2}C_{a}*\right)}{N*}\right)S*-\sigma A*+\mu S*(\sigma +\gamma +\nu_{1})=\left(\frac{\beta \left(A*+\alpha_{1}C_{u}*+\alpha_{2}C_{a}*\right)}{AN*}\right)S*(\nu_{2}+\mu +d_{c})=\frac{\gamma A*}{C_{u}*}\left(\delta +\mu +d_{c}\right)=\frac{\nu_{2}C_{u}*}{C_{a}*}+\frac{\nu_{1}A*}{C_{a}*}(\omega +\mu )=\frac{\delta C_{a}*}{T_{c}*}\omega =\frac{\mu R*}{T_{c}*}$$ (31)

$$\frac{\mathrm{dL}}{\mathrm{dt}}=\left(1-\frac{S^{*}}{S}\right)\left[\left(\frac{\beta \left(A*+\alpha_{1}C_{u}*+\alpha_{2}C_{a}*\right)}{N*}\right)S*-\mathrm{\sigma A}*+\mathrm{\mu S}*-\left(\frac{\beta \left(A+\alpha_{1}C_{u}+\alpha_{2}C_{a}\right)}{N}\right)S+\mathrm{\sigma A}-\mathrm{\mu S}\right]+\left(1-\frac{A^{*}}{A}\right)\left[\left(\frac{\beta \left(A+\alpha_{1}C_{u}+\alpha_{2}C_{a}\right)}{N}\right)S-\left(\frac{\beta \left(A*+\alpha_{1}C_{u}*+\alpha_{2}C_{a}*\right)}{\text{AN}*}\right)S*A\right]+\left(1-\frac{{C_{u}}^{*}}{C_{u}}\right)\left[\mathrm{\gamma A}-\frac{\mathrm{\gamma A}*}{C_{u}*}C_{u}\right]+\left(1-\frac{{C_{a}}^{*}}{C_{a}}\right)\left[\nu_{2}C_{u}+\nu_{1}A-\frac{\nu_{2}C_{u}*}{C_{a}*}+\frac{\nu_{1}A*}{C_{a}*}C_{a}\right]+\left(1-\frac{{T_{c}}^{*}}{T_{c}}\right)\left[\delta C_{a}-\frac{\delta C_{a}*}{T_{c}*}T_{c}\right]+\left(1-\frac{R^{*}}{R}\right)\left[\frac{\mathrm{\mu R}*}{T_{c}*}T_{c}-\mathrm{\mu R}\right]=\left(1-\frac{S^{*}}{S}\right)\left[\frac{\mathrm{\beta A}*S*}{N*}+\frac{\beta \alpha_{1}C_{u}*S*}{N*}+\frac{\beta \alpha_{2}C_{a}*S*}{N*}-\mathrm{\sigma A}*+\mathrm{\mu S}*-\frac{\mathrm{\beta AS}}{N}-\frac{\beta \alpha_{1}C_{u}S}{N}-\frac{\beta \alpha_{2}C_{a}S}{N}+\mathrm{\sigma A}-\mathrm{\mu S}\right]+\left(1-\frac{A^{*}}{A}\right)\left[\frac{\mathrm{\beta AS}}{N}-\frac{\mathrm{\beta AS}*}{N*}+\frac{\beta \alpha_{1}C_{u}S}{N}-\frac{\beta \alpha_{1}C_{u}*S*A}{A*N*}+\frac{\beta \alpha_{2}C_{a}S}{N}-\frac{\beta \alpha_{2}C_{a}*S*A}{A*N*}\right]+\left(1-\frac{{C_{u}}^{*}}{C_{u}}\right)\mathrm{\gamma A}\left[1-\frac{A*C_{u}}{AC_{u}*}\right]+\left(1-\frac{{C_{a}}^{*}}{C_{a}}\right)\left[\nu_{2}C_{u}\left(1-\frac{{C_{u}}^{*}C_{a}}{C_{u}C_{a}*}\right)+\nu_{1}A\left(1-\frac{A^{*}C_{a}}{AC_{a}*}\right)\right]+\delta C_{a}\left(1-\frac{{T_{c}}^{*}}{T_{c}}\right)\left[1-\frac{C_{a}*T_{c}}{C_{a}T_{c}*}\right]-\mathrm{\mu R}\left(1-\frac{R^{*}}{R}\right)\left[1-\frac{R*T_{c}}{{\mathrm{RT}}_{c}*}\right]$$ (32)

$$=\left(1-\frac{S^{*}}{S}\right)\left[-\frac{\mathrm{\beta AS}}{N}\left(1-\frac{A*S*N}{\mathrm{ASN}*}\right)-\frac{\beta \alpha_{1}C_{u}S}{N}\left(1-\frac{C_{u*}S*N}{C_{u}\mathrm{SN}*}\right)+\frac{\beta \alpha_{2}C_{a}S}{N}\left(1-\frac{C_{a*}S*N}{C_{a}\mathrm{SN}*}\right)-\mathrm{\sigma A}\left(1-\frac{A^{*}}{A}\right)-\mathrm{\mu S}-\left(1-\frac{S^{*}}{S}\right)\right]+\left(1-\frac{A^{*}}{A}\right)\left[\frac{\mathrm{\beta AS}}{N}\left(1-\frac{A*S*N}{\mathrm{ASN}*}\right)-\frac{\beta \alpha_{1}C_{u}S}{N}\left(1-\frac{C_{u*}S*\text{AN}}{C_{u}\mathrm{SA}*N*}\right)+\frac{\beta \alpha_{2}C_{a}S}{N}\left(1-\frac{C_{a*}S*\text{AN}}{C_{a}\mathrm{SA}*N*}\right)\right]+\left(1-\frac{{C_{u}}^{*}}{C_{u}}\right)\mathrm{\gamma A}\left[1-\frac{A*C_{u}}{AC_{u}*}\right]+\left(1-\frac{{C_{a}}^{*}}{C_{a}}\right)\left[\nu_{2}C_{u}\left(1-\frac{{C_{u}}^{*}C_{a}}{C_{u}C_{a}*}\right)+\nu_{1}A\left(1-\frac{A^{*}C_{a}}{AC_{a}*}\right)\right]+\delta C_{a}\left(1-\frac{{T_{c}}^{*}}{T_{c}}\right)\left[1-\frac{C_{a}*T_{c}}{C_{a}T_{c}*}\right]-\mathrm{\mu R}\left(1-\frac{R^{*}}{R}\right)\left[1-\frac{R*T_{c}}{{\mathrm{RT}}_{c}*}\right]\;$$ (33)

$$=-\mathrm{\mu S}{\left(1-\frac{S^{*}}{S}\right)}^{2}-\frac{\mathrm{\beta AS}}{N}\left(1-\frac{S^{*}}{S}\right)\left(1-\frac{A^{*}S^{*}N}{\mathrm{ASN}*}\right)-\frac{\beta \alpha_{1}C_{u}S}{N}\left(1-\frac{S^{*}}{S}\right)\left(1-\frac{C_{u*}S*N}{C_{u}\mathrm{SN}*}\right)-\frac{\beta \alpha_{2}C_{a}S}{N}\left(1-\frac{S^{*}}{S}\right)\left(1-\frac{C_{a*}S*N}{C_{a}\mathrm{SN}*}\right)-\mathrm{\sigma A}\left(1-\frac{S^{*}}{S}\right)\left(1-\frac{A^{*}}{A}\right)+\frac{\mathrm{\beta AS}}{N}\left(1-\frac{A^{*}}{A}\right)\left(1-\frac{S^{*}N}{\mathrm{SN}*}\right)+\frac{\beta \alpha_{1}C_{u}S}{N}\left(1-\frac{A^{*}}{A}\right)\left(1-\frac{C_{u*}S*\text{AN}}{C_{u}\mathrm{SA}*N*}\right)+\frac{\beta \alpha_{2}C_{a}S}{N}\left(1-\frac{A^{*}}{A}\right)\left(1-\frac{C_{a*}S*\text{AN}}{C_{a}\mathrm{SA}*N*}\right)+\mathrm{\sigma A}\left(1-\frac{{C_{u}}^{*}}{C_{u}}\right)\left(1-\frac{A^{*}C_{u}}{AC_{u}*}\right)+\nu_{2}C_{u}\left(1-\frac{{C_{a}}^{*}}{C_{a}}\right)\left(1-\frac{{C_{u}}^{*}C_{a}}{C_{u}C_{a}*}\right)+\nu_{1}A\left(1-\frac{{C_{a}}^{*}}{C_{a}}\right)\left(1-\frac{A^{*}C_{a}}{AC_{a}*}\right)+\delta C_{a}\left(1-\frac{{T_{c}}^{*}}{T_{c}}\right)\left(1-\frac{C_{a}*T_{c}}{C_{a}T_{c}*}\right)-\mathrm{\mu R}\left(1-\frac{R^{*}}{R}\right)\left(1-\frac{R*T_{c}}{{\mathrm{RT}}_{c}*}\right)$$

$$=-\mathrm{\mu S}{\left(1-\frac{S^{*}}{S}\right)}^{2}+P_{1}\left(S,A,C_{a},C_{u},T_{c},R\right)+P_{2}\left(S,A,C_{a},C_{u},T_{c},R\right)$$ (34)

where,

$$P_{1}\left(S,A,C_{a},C_{u},T_{c},R\right)=-\frac{\mathrm{\beta AS}}{N}\left(1-\frac{S^{*}}{S}\right)\left(1-\frac{A^{*}S^{*}N}{\mathrm{ASN}*}\right)-\frac{\beta \alpha_{1}C_{u}S}{N}\left(1-\frac{S^{*}}{S}\right)\left(1-\frac{C_{u*}S*N}{C_{u}\mathrm{SN}*}\right)-\frac{\beta \alpha_{2}C_{a}S}{N}\left(1-\frac{S^{*}}{S}\right)\left(1-\frac{C_{a*}S*N}{C_{a}\mathrm{SN}*}\right)-\mathrm{\sigma A}\left(1-\frac{S^{*}}{S}\right)\left(1-\frac{A^{*}}{A}\right)-\mathrm{\mu R}\left(1-\frac{R^{*}}{R}\right)\left(1-\frac{R*T_{c}}{{\mathrm{RT}}_{c}*}\right)$$

$$P_{2}\left(S,A,C_{a},C_{u},T_{c},R\right)=\frac{\mathrm{\beta AS}}{N}\left(1-\frac{A^{*}}{A}\right)\left(1-\frac{S^{*}N}{\mathrm{SN}*}\right)+\frac{\beta \alpha_{1}C_{u}S}{N}\left(1-\frac{A^{*}}{A}\right)\left(1-\frac{C_{u*}S*\text{AN}}{C_{u}\mathrm{SA}*N*}\right)+\frac{\beta \alpha_{2}C_{a}S}{N}\left(1-\frac{A^{*}}{A}\right)\left(1-\frac{C_{a*}S*\text{AN}}{C_{a}\mathrm{SA}*N*}\right)+\mathrm{\sigma A}\left(1-\frac{{C_{u}}^{*}}{C_{u}}\right)\left(1-\frac{A^{*}C_{u}}{AC_{u}*}\right)+\nu_{2}C_{u}\left(1-\frac{{C_{a}}^{*}}{C_{a}}\right)\left(1-\frac{{C_{u}}^{*}C_{a}}{C_{u}C_{a}*}\right)+\nu_{1}A\left(1-\frac{{C_{a}}^{*}}{C_{a}}\right)\left(1-\frac{A^{*}C_{a}}{AC_{a}*}\right)+\delta C_{a}\left(1-\frac{{T_{c}}^{*}}{T_{c}}\right)\left(1-\frac{C_{a}*T_{c}}{C_{a}T_{c}*}\right)$$

$P_{1}\leq 0\;$ whenever

$$\mathrm{ASN}*\geq A^{*}S^{*}N,C_{u}\mathrm{SN}*\geq {C_{u}}^{*}S^{*}N,C_{a}\mathrm{SN}*\geq {C_{a}}^{*}S^{*}N,RT_{c}*\geq R*T_{c}$$ (35)

and $P_{2}\leq 0\;$ whenever

$$S^{*}N\geq \mathrm{SN}*,C_{u}*S*\text{AN}\geq C_{u}\mathrm{SA}*N*,{C_{a}}^{*}S^{*}\text{AN}\geq C_{a}\mathrm{SA}*N*,A*C_{u}\geq AC_{u}*,C_{u}*C_{a}\geq C_{u}{C_{a}}^{*},A*C_{a}\geq A{C_{a}}^{*},{C_{a}}^{*}T_{c}\geq C_{a}T_{c}*$$ (36)

Thus, $\frac{\mathrm{dL}}{\mathrm{dt}}\leq 0$ if the condition in (35) and (36) holds.

therefore, by LaSalle asymptotic stability theorem ( LaSalle, 1976), and Oke *et al.* (2020) the positive equilibrium state $\frac{\mathrm{dL}}{\mathrm{dt}}$ is globally asymptotically stable in the positive region $\;R_{+}^{6}$ .

## 3. Numerical computation

The numerical study is carried out using maple software embedded code for the Runge-Kutta of fourth order. Here, the subsequent default values are assumed for the embedded parameters taken from theoretical studies in literatures $\gamma =0.9,\beta =0.008,\sigma =0.59,d\left(c\right)=0.00693,\mu =0.00693,\omega =0.1,\nu_{1}=0.002,\nu_{2}=0.002,\alpha_{1}=0.0016,\alpha_{2}=0.0016,\delta =0.0085,\Pi =0.07$ . The values remain unchanged throughout* the computations except otherwise indicated.

**Figure 2.**
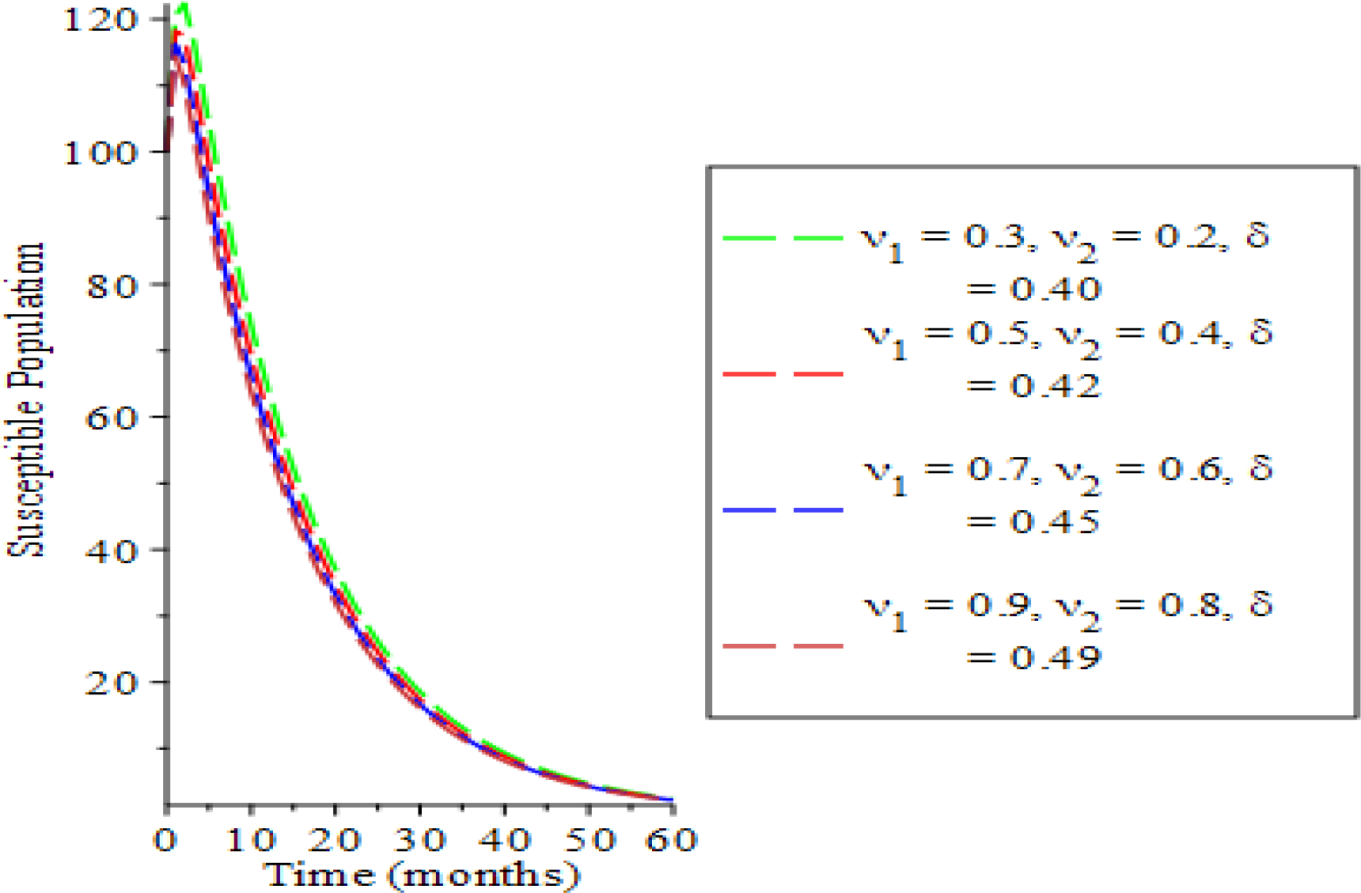
Behavioral dynamics of susceptible population when varying testing rate for acute and chronic individuals and treatment for chronic individuals.

**Figure 3.**
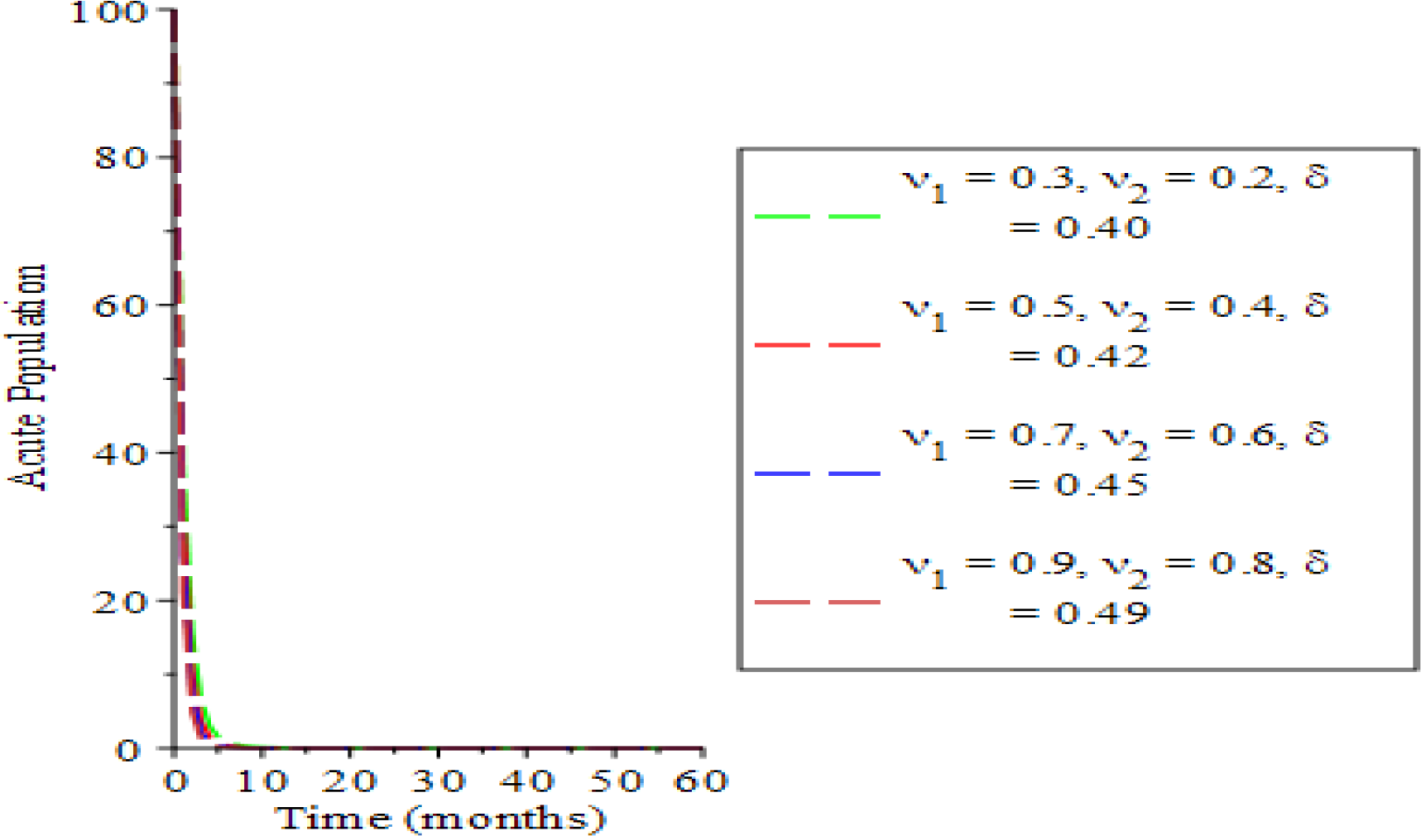
Behavioral dynamics of acute population when varying testing rate for acute and chronic individuals and treatment for chronic individuals.

**Figure 4.**
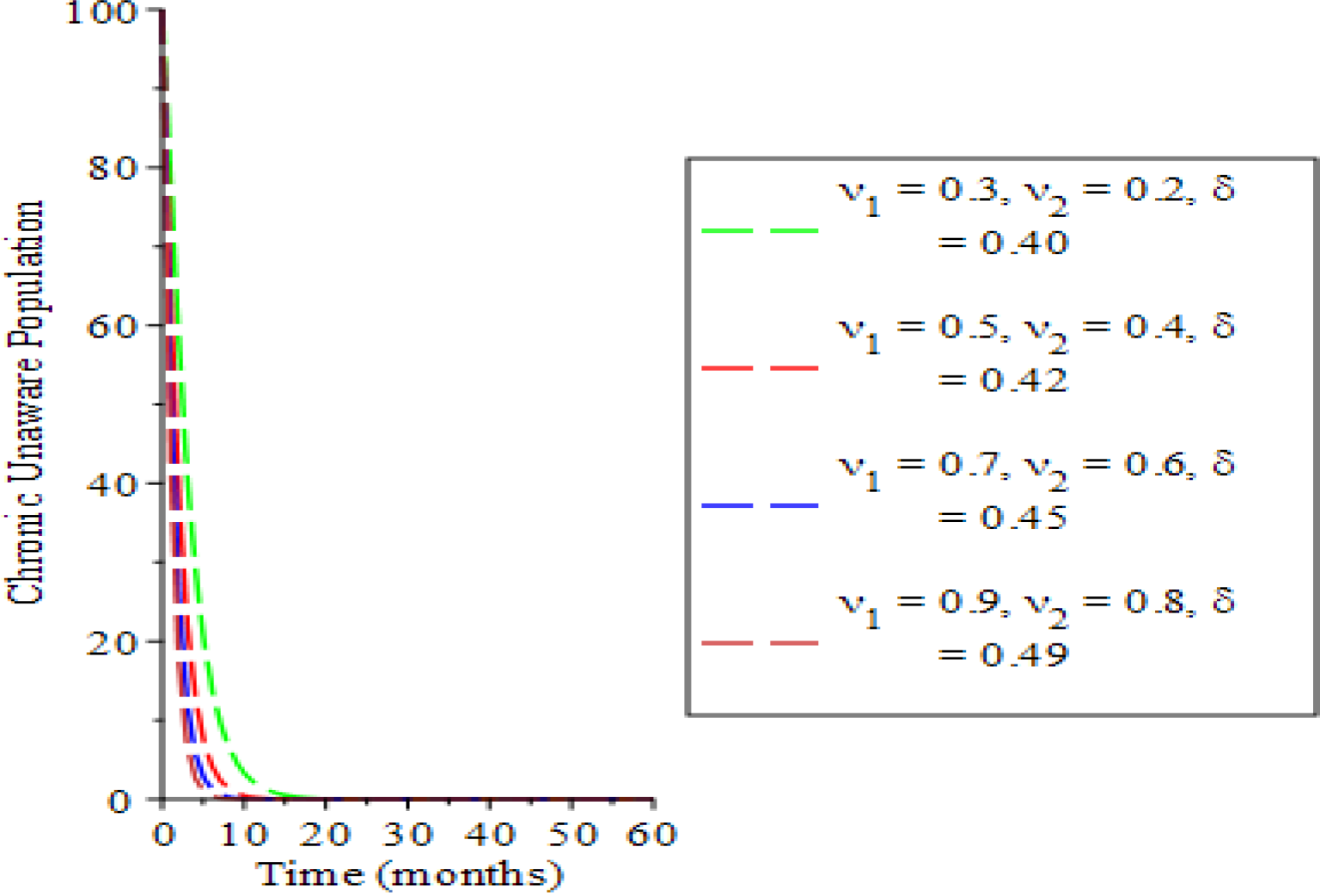
Behavioral dynamics of chronic unaware population when varying testing rate for acute and chronic individuals and treatment for chronic individuals.

**Figure 5.**
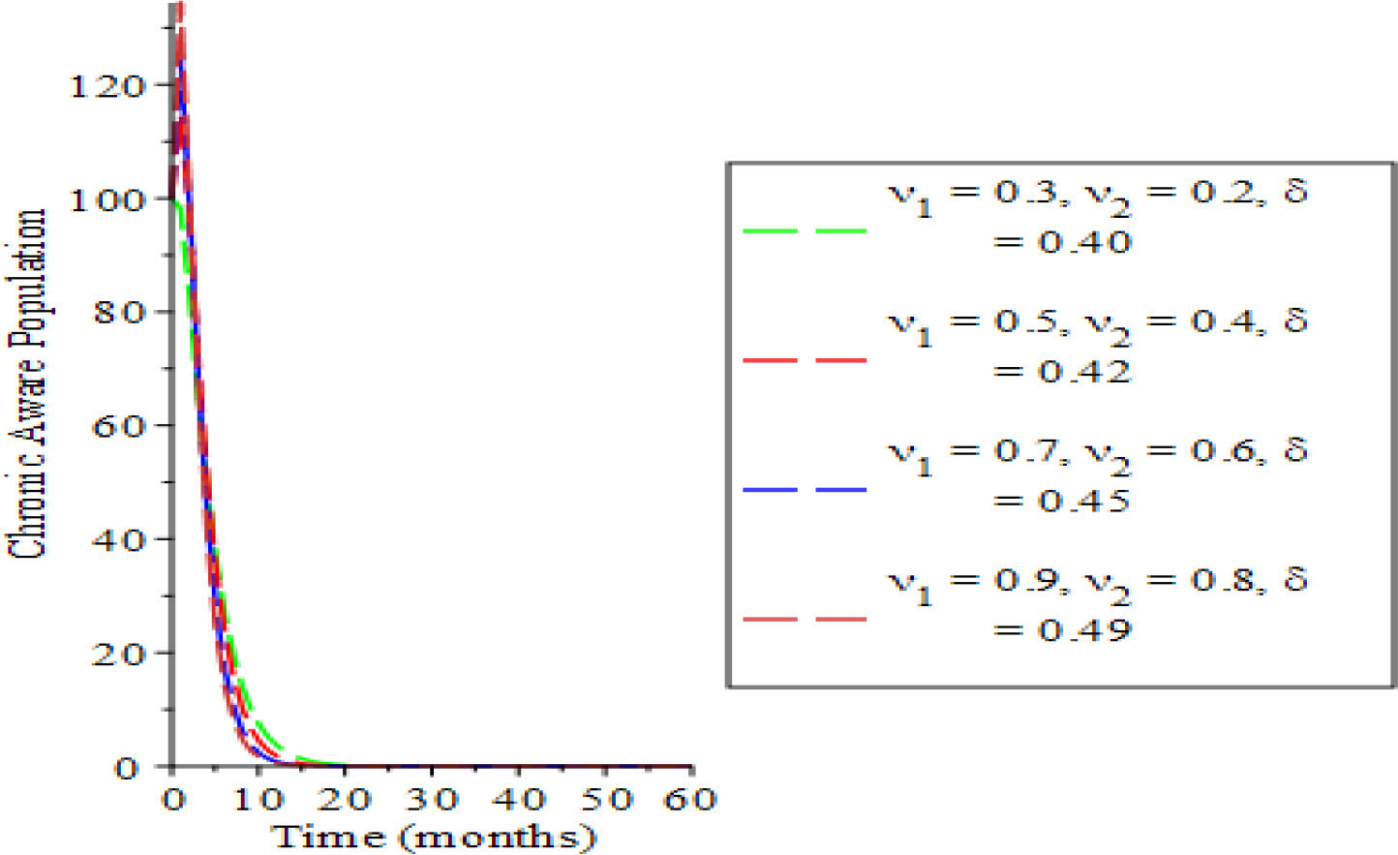
Behavioral dynamics of chronic aware population when varying testing rate for acute and chronic individuals and treatment for chronic individuals.

**Figure 6.**
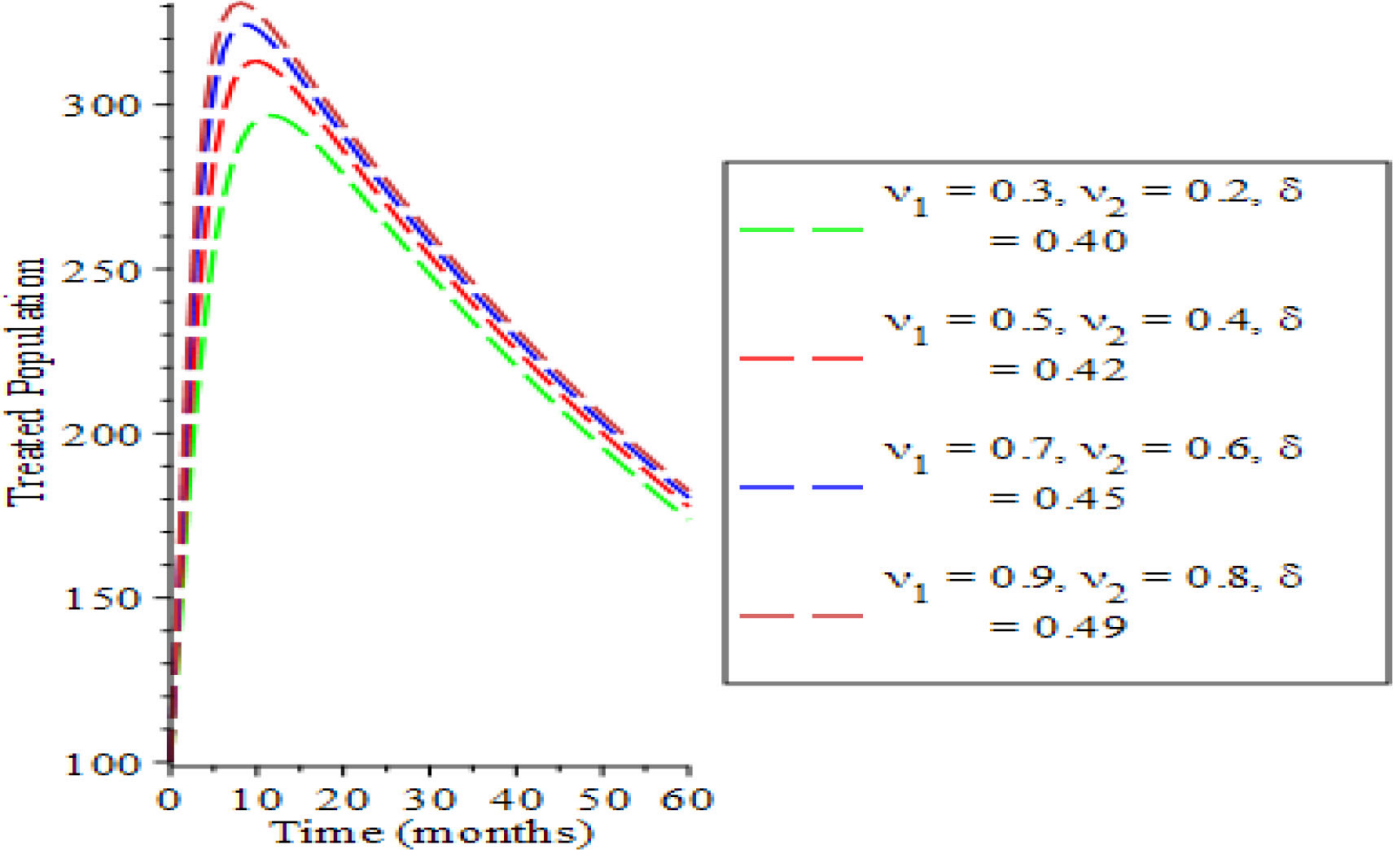
Behavioral dynamics of treated population when varying testing rate for acute and chronic individuals and treatment for chronic individuals.

**Figure 7.**
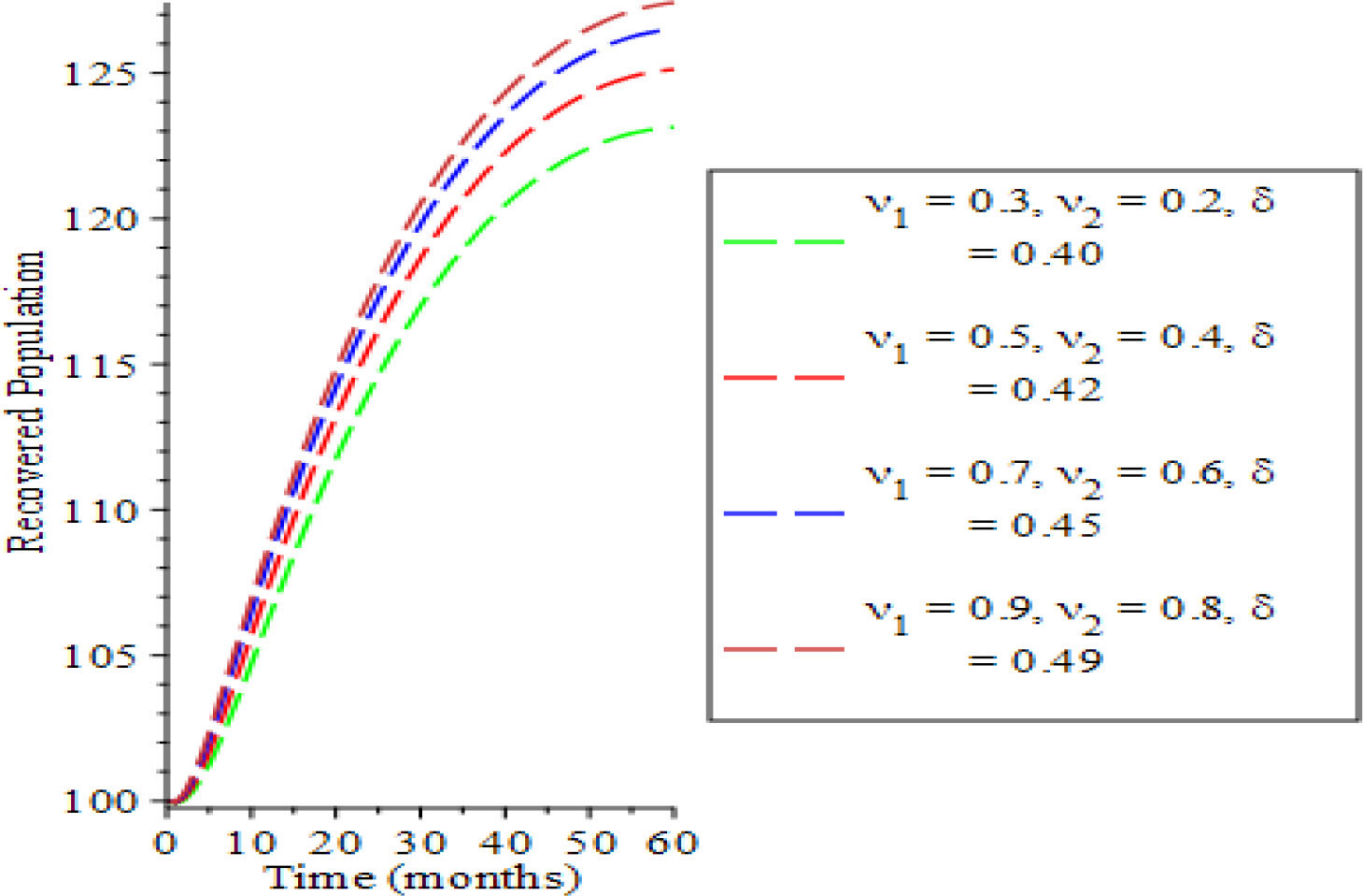
Behavioral dynamics of recovered population when varying testing rate for acute and chronic individuals and treatment for chronic individuals.

The effects of varying the testing rate of the acute individuals $\left(\upsilon_{1}\right),$ testing rate of chronic individuals ( $\upsilon_{2}$ ) and treatment rate of chronic individuals ( δ ) on the population dynamics are shown in Figures 2 to 7. From Figures 2 and 3, an increase in the parameters values reduces susceptible and acute populations thereby reducing the spread HBV due to low interaction between the host immune system and the virus. Therefore, the appearance of HBV and the pathogenesis reduces, which in so doing, lessens the potential injury on the liver. Hence, the liver is shielded from hepatocellular carcinoma over time. The rate of chronic unaware and chronically aware individuals is examined in Figures 4 and 5. The parameter variations show a significant decrease in the chronic unaware population which implies that testing at that stage is a great tool for reducing the disease transmission. The transmission process dies down as the time progresses; this discourages liver inflammation as a result of lowering the infected individuals. Meanwhile, the chronic population in Figure 5, depicts a high significant influence of the acutely infected and chronically unaware infected individuals over time. A chronic infection phase is found at the time range 10<t<20, as such, the individuals are exposed to liver carcinoma or cirrhosis. Hence, the chronic population diminishes as the parameters are increased.

In Figures 6 and 7, the impact of varying the testing rate of the acute individuals $\left(\upsilon_{1}\right),$ testing rate of chronic individuals ( $\upsilon_{2}$ ) and treatment rate of chronic individuals ( δ ) on the treated and the recovered population are presented. The treated population increases with parameters variation along the rising time ( *t*) as a result of long time effect of parameter values. The recovery rate is enhanced as observed in Figure 7 due to significant simulation of surface antibodies of Hepatitis B. This is in conformity with the works of Pang *et al*. (2010) and Ullah *et al*. (2019). This result implies that an intensification in testing at all infectious states and rise in treatment of chronic individual will bring about a reduction in the HBV transmission process which is a response to the WHO goal for 2030 that concentrating efforts on awareness program and campaign will sure bring about a decrease or eradication in the transmission process of the virus ( WHO, 2020).

## 4. Conclusion

A deterministic model of hepatitis B testing was developed and investigated, which included testing in the chronic unaware state as well as testing in all infectious states. The model has disease-free and endemic equilibria. The basic reproduction number was calculated using the next generation matrix method. The equilibria's local and global stability were discussed and shown to be asymptotically stable. The testing and treatment rate effects were thoroughly discussed.

## Data availability

No data are associated with this article.

## Grant information

None declared.

## Competing interests

None declared.
