# Supplementary material for: The impact of testing and treatment on the dynamics of Hepatitis B virus
Source: F1000Res. 2021 Sep 17;10:936. [Version 1] doi: 10.12688/f1000research.72865.1 (PMC8686325; doi:10.12688/f1000research.72865.1)
Supplement: Peer review history

# Peer review: Reviewer response for version 1

**DOI:** 10.5256/f1000research.76473.r94650

**Published:** 2021-12-17

**Authors:** Segun oke

**Affiliations:** 1 Department of Mathematical and Applied Mathematics, University of Pretoria, Pretoria, South Africa

The authors worked on the impact of testing and treatment on the dynamics of Hepatitis B virus. Although the manuscript is well presented and gives new insight into the Hepatitis B virus, especially for unvaccinated men who have sex with men (MSM) and heterosexual people who have multiple sexual partners or have contact with sex workers.

 In their paper, a mathematical model was formulated to analyse the acquisition and transmission process of the virus with the view of identifying the possible way of reducing the menace and mitigating the risk of the virus. The models’ positivity and boundedness were demonstrated using well-known theorems. Equating the differential equations to zero demonstrates the equilibria of the solutions i.e., the disease-free and endemic equilibrium. The next Generation Matrix method was used to compute the basic reproduction number for the models. Local and global stabilities of the models were shown via linearization and contrition of Lyapunov function methods respectively. The authors concluded by showing disease-free, endemic equilibria. The basic reproduction number was calculated using the next-generation matrix method. The equilibria's local and global stability were discussed and shown to be asymptotically stable. The testing and treatment rate effects were thoroughly discussed. I believe that I have an appropriate level of expertise to confirm that it is of an acceptable scientific standard.

**Some minor comments: **

1. The authors need to review more literature to back up their claims. 
2. The introduction is too short and is not well cited. For example:
*"In highly endemic areas, the most common form of transmission of hepatitis B is from mother to child at birth (vertical transmission) or through horizontal route (contact with infected blood), particularly from infected children to uninfected children during the first five years of life. vaginal and spermatic fluids and, to a lesser degree, perspiration, breast milk, tears, and urine. In particular, hepatitis B can be transmitted through sexual contact in unvaccinated men who have sex with men (MSM) and heterosexual people who have multiple sexual partners or have contact with sex workers"* there is no citation here.
3. The authors need to add epidemiological interpretation and recommendations to the manuscript.
4. The conclusion needs to be rewritten. 

Is the work clearly and accurately presented and does it cite the current literature?

No

If applicable, is the statistical analysis and its interpretation appropriate?

Not applicable

Are all the source data underlying the results available to ensure full reproducibility?

Yes

Is the study design appropriate and is the work technically sound?

Yes

Are the conclusions drawn adequately supported by the results?

No

Are sufficient details of methods and analysis provided to allow replication by others?

Yes

Reviewer Expertise:

Mathematical modelling, Mathematical Oncology, Optimal control, Epidemiology, etc

I confirm that I have read this submission and believe that I have an appropriate level of expertise to confirm that it is of an acceptable scientific standard.

# Peer review: Reviewer response for version 1

**DOI:** 10.5256/f1000research.76473.r94651

**Published:** 2021-10-06

**Authors:** Sulyman Olakunle Salawu

**Affiliations:** 1 Department of Mathematics, Landmark University, Omu-aran, Nigeria

Reviewer Reports on the impact of testing and treatment on the dynamics of Hepatitis B virus.

 The impacts of different parameters were examined. The manuscript is well written and presented logically. The abstract captures accurately the whole work and the introduction is sound. However, I have the following comments and queries for the authors:

1. The authors should explain what prompted the choice of method of solution used over other techniques.
2. The significance and motivation of the study are not clear. The authors should clarify these.
3. There is a need to reference equation (3) to establish its correctness and source.
4.  Some grammatical mistakes should be checked through.
5. Why have the authors chosen to engage in this study?
6. A likely extension of the study should be stated or recommended. 

The manuscript should be indexed subject to minor correction.

Is the work clearly and accurately presented and does it cite the current literature?

Yes

If applicable, is the statistical analysis and its interpretation appropriate?

Not applicable

Are all the source data underlying the results available to ensure full reproducibility?

No source data required

Is the study design appropriate and is the work technically sound?

Yes

Are the conclusions drawn adequately supported by the results?

Yes

Are sufficient details of methods and analysis provided to allow replication by others?

Yes

Reviewer Expertise:

Mathematical Modeling and flow dynamic

I confirm that I have read this submission and believe that I have an appropriate level of expertise to confirm that it is of an acceptable scientific standard.
